# Tropical Medicine

**Published:** 1907-09-07

**Authors:** 


### TROPICAL MEDICINE.

The recent advances in our knowledge of tropical diseases have rendered it imperative that special teaching by experts should be given to young graduates about to take up their different spheres of work in the various colonies. Thus have arisen, in most of the countries that possess tropical dependencies, special schools of training usually designated Schools of Tropical Medicine. In dealing with the general question of the value of instruction in tropical medicine we may divide graduates into three classes: (1) Those who do not intend to practise abroad, *i.e.*, who will spend their life in England; (2) those who intend to practise abroad and who are leaving England for the first time; (3) and those who have resided and practised in the tropics for longer or shorter periods, and who have had either (*a*) special instruction before going abroad; or (*b*) no such instruction. Each of these classes must necessarily vary greatly from the others in its needs, and it will conduce to clearness, therefore, to discuss them separately.

### What It Is.

Before, however, commencing this discussion let us define what tropical medicine really means, and how it differs clinically and practically from the general medicine taught in the ordinary medical schools. As regards the first, the clinical aspect, there is not much to say; the methods employed are the same, but in the tropics, besides almost all the ordinary diseases of temperate climates, there are some peculiar forms, which are difficult to see and study at home.

### Practical Aspects of Tropical Medicine.

As regards the second, the practical aspect of tropical medicine, we find much the same applies; the methods are again similar, the blood has to be examined in just the same way, bacteria have to be grown and studied, and so on. Where the difference comes in is that certain tropical diseases have as their causative agents peculiar parasites, often of a protozoal nature. Further, besides an elementary knowledge of protozoology, helminthology, and entomology must be mastered by the would-be tropical expert, and this alone at once differentiates the scientific medicine of temperate climates from that of the hotter regions. This blending of the study of pure zoology with that of medicine is the most striking feature that has occurred in medicine within recent years. It has resulted in the production of voluminous volumes on mosquitoes and other insects previously hardly known or mentioned, and it has thrown fresh light and interest on the complicated study of the protozoa, raising that subject to a height of interest almost exceeding that of bacteriology. This of itself is sufficient to indicate a necessity of special instruction, and it places the practical side of tropical medicine at least on a plane with special subjects such as bacteriology, in which, of course, post-graduate teaching is often required.

### Its Value to the Graduate.

Having explained, then, what the term tropical medicine means, we may return to the question of its value for the three classes of graduates, mentioned at the beginning of the paper. We have, taking them in the same order (1) graduates who do not intend to practise abroad, *i.e.*, who will spend their lives in England. Do these require a special course of instruction in tropical medicine? It may at once be said that they do not, because an ordinary general practitioner sees only an odd case or two of tropical disease in the course of his life. Further, it must be remembered that a considerable amount of teaching is carried out on this subject in the ordinary medical and pathological courses given to students, and this is quite sufficient for the majority. What good, for example, would it be for a doctor, say, in some country district, to be specially coached up as a young graduate in the differences between a culex and an anopheles mosquito, or how to tell a tsetse fly from a tabanus, when he would never encounter such an insect for the rest of his life? There is, however, another class of graduate: young men, keen on keeping up-to-date in all the recent scientific work, who are reading for higher examinations, or have ambitions to get on the staff of a hospital; to them a special course of instruction in tropical medicine will prove not only interesting, but useful, as they may in their future consulting work find knowledge of tropical diseases, parasites, and infecting agents of great advantage, and it will certainly prevent them from falling into the ludicrous and serious mistakes that are so often perpetrated by leading authorities in dealing with cases from abroad. So many invalids are constantly coming from our colonial possessions to London that it is almost essential for the ordinary consultant to know something about tropical medicine, as they often find their way to him, even though there are now special consultants on tropical diseases.

### Its Value for the Colonial Practitioner.

When we turn to our second class, "graduates who intend to practise abroad and who are leaving England for the first time," then the answer to the question, "Do they require a special course of instruction in tropical medicine?" is a very simple one: it is "Yes, emphatically they do." The young graduate, let him be clever or the reverse, let him have assimilated all the teaching bearing upon tropical diseases he has had placed before him as a student, let him have mastered blood diseases and bacteriology, still requires the special instruction given at schools of tropical medicine before he sets out for abroad. If he belong to the category of the clever, he will quickly pick up the new work; he will see some clinical cases (a point to be returned to later), and, armed with his microscope, at the end of his training he will be competent to give an accurate diagnosis on at least the commoner forms of tropical disease. If he belong to the category of the backward, still more is it essential that he should be trained, drilled, and coached before he is set at liberty to handle the lives of white people abroad, because, as so often happens there, he is absolutely alone, and has no one to turn to to advise or assist him. It is hardly necessary to emphasise this point further. The Navy, the Army, the Foreign Office, and the Colonial Office all insist now that their medical officers shall have instruction in tropical diseases before they take up their appointments, and this clearly shows the value they set on such instruction. The Army and Navy have their own schools of instruction, Colonial medical men are sent to the schools of tropical medicine at London or Liverpool, and these also afford facilities to private students, missionaries, and men taking up posts such as railways, mines, or other similar situations afford. Most of these latter companies now will give the preference to a medical man who can produce a certificate of a course of instruction from one or the other of the schools, and much the same applies in Germany, France, Portugal, and other Continental countries.

### The London School of Tropical Medicine.

It may interest intending applicants for Colonial or other posts in the Tropics if one gives a short *resumé* of what the course of instruction at the London School of Tropical Medicine consists of, and how long it lasts. The sessions extend over three months each, and there are three a year; the Colonial Office requires that men selected for posts in the tropics should attend one such session, and, in addition to exhibiting regularity in attendance, should pass the examination at the end of the course. Failure to do so involves loss of the appointment. Other students may take the examination or not, as they choose, and, if successful, get a certificate to that effect. The courses qualify, and are useful preparations, for the Diploma of Tropical Medicine and Hygiene granted by the University of Cambridge. It has been argued that three months is not sufficient time to train men in, and six months' courses have been suggested. There are difficulties in the way for such extension, especially in the case of men on leave from abroad who are desirous of taking out a session, and, after all, as the student is required to attend from 10 to 4 or 5 daily during the three months, this is really equivalent to an ordinary two hours' course of six months. The instruction is divided into three headings: (1) the practical work in the laboratory, by far the most important and useful of the three; (2) clinical instruction in the wards and the demonstration of cases; and (3) systematic lectures. Under the first heading the student is trained in hæmatology, protozology, bacteriology, entomology, helminthology, pathology, and morbid anatomy; the tuition is eminently practical, the student preparing, staining, and mounting his own specimens, so that when he completes his studies he has in possession a very complete set of specimens, which serve him as standards for the future.

Under the second heading comes the clinical instruction in the wards: the paucity of cases is a serious drawback in this part of the teaching, though, of course, one case studied thoroughly is much more important than many simply glanced at, but still at the same time this is a real defect, and one which it is difficult to remedy. In other ways also more use might be made of the clinical material, because after all the majority of the men who go abroad go as general practitioners who have to make their living at practice, not as scientific specialists who go in the hope of making some far-reaching discovery that will startle the world.

Under the third heading, "Systematic lectures," little need be said. They are too numerous, and are not of much use, because most of the work covered in them has already been thoroughly gone into in the laboratory in the practical course. Having shown, then, the extreme value and necessity for such a course for all graduates who may possibly be called upon to follow their profession outside these islands we may pass on to our next class—(3) graduates who have resided and practised in the tropics for longer and shorter periods, and who have had either (*a*) special instruction in tropical diseases before going abroad, or (*b*) no such instruction. For convenience let us discuss (*b*) first. There are many of those, some in the Colonial service, others in private practice, who on their return home on leave desire to brush themselves up in recent work. It will entirely depend, of course, on what they want to see and do. Some may want to brush up their surgery, others their special subjects, such as ophthalmology or diseases of the throat, while others, interested in blood and microscopical work, may find it more useful to attend one of the tropical schools to get the special work taught there—work which was in its infancy more or less when they left those islands in their youth. There are many varieties, therefore, in this class, and each must judge for himself what is best for him to do. Under heading (*a*) we now have numerous graduates, who have passed through the tropical schools or naval and army courses of instruction and are returning home on leave from time to time. Here again it will depend on what the individual's speciality is abroad. To this class the tropical schools will not appeal. What they require is post-graduate work of various sorts, and various institutions where the post-graduate can get short courses on surgery, medicine, recent research on tropical medicine, bacteriology, hæmatology, the special senses, *x*-ray study, with plenty of clinical and pathological work, supply this need, and, as a result, are much favoured by this special class of graduate.

Our last variety of graduate is the man with scientific leanings who comes home on leave, and wishes perchance to devote himself to some special branch of science or some special disease. Few places are open to him, but Liverpool, or rather the Liverpool School of Tropical Medicine, will receive him willingly, and, if he has ability, will probably get him a post on one of their numerous expeditions. A vast amount of research has been accomplished there in addition to the ordinary tropical courses for the younger graduate, and any man from the tropics who wishes a helping hand in this direction cannot do better than go there.

